# Carbon Dioxide Insufflation for Assessment of Pericardial Adhesions and Informed Decision‐Making for Percutaneous Epicardial Access in a Patient With Prior Cardiac Surgery

**DOI:** 10.1002/joa3.70348

**Published:** 2026-04-20

**Authors:** Yuhei Kasai, Takayuki Kitai, Junji Morita, Gentaku Hama, Kennosuke Yamashita

**Affiliations:** ^1^ Department of Cardiology, Sapporo Heart Center Sapporo Cardiovascular Clinic Sapporo Japan; ^2^ Department of Cardiac Surgery, Sapporo Heart Center Sapporo Cardiovascular Clinic Sapporo Japan; ^3^ Heart Rhythm Center, Department of Cardiovascular Medicine Sendai Kosei Hospital Sendai Miyagi Japan

**Keywords:** catheter ablation, CO_2_ insufflation, epicardial access, pericardial adhesions, ventricular tachycardia

## Abstract

Carbon dioxide insufflation via intentional coronary sinus perforation allowed real‐time assessment of pericardial adhesion distribution before pericardial puncture. Despite localized adhesions, visualization of preserved pericardial space at the intended access site enabled informed procedural planning and safe percutaneous epicardial ablation in a patient with prior cardiac surgery.

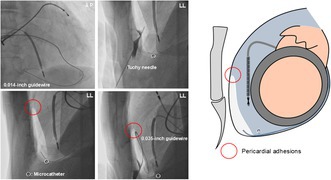

Percutaneous epicardial access is used for ventricular tachycardia (VT) ablation, but its safety may be compromised by pericardial adhesions, which are a predictor of access‐related complications [1]. Intentional coronary sinus (CS) exit and carbon dioxide (CO_2_) insufflation have recently been introduced to improve procedural safety [2, 3], but the presence and distribution of adhesions often remain unknown until puncture is attempted.

While prior studies have focused on CO_2_ insufflation primarily as a means to facilitate pericardial access by increasing separation within the pericardial space, its potential role in pre‐procedural strategic decision‐making remains underappreciated. We herein report a case in which CO_2_ insufflation was used not only to aid epicardial access but also to visualize gas dispersion within the pericardial space, allowing pre‐puncture assessment of pericardial adhesions and informed procedural planning. In this case, CO_2_ insufflation was employed in a patient with a history of surgical excision of a left atrial myxoma, which directly informed the decision to proceed with a percutaneous epicardial ablation.

A 60‐year‐old man with ischemic cardiomyopathy had undergone implantation of a non‐MRI‐conditional cardiac resynchronization therapy defibrillator (CRT‐D; Boston Scientific, Marlborough, MA, USA) 14 years ago. Three years ago, he underwent endocardial‐only VT ablation targeting mid‐diastolic potentials at the septal left ventricular (LV) apex. One year before presentation, computed tomography identified an LA myxoma, which was successfully resected via a robot‐assisted minimally invasive right thoracic approach (Figure 1A,B). The main working port was placed in the fourth intercostal space, followed by a longitudinal pericardiotomy anterior to the phrenic nerve to expose the LA for tumor excision. Despite amiodarone therapy, frequent CRT‐D therapies for recurrent VT occurred postoperatively. Noninvasive programmed stimulation induced VT with QRS features suggestive of an epicardial exit at the LV apex (Figure 1C), which met established electrocardiographic criteria for an epicardial origin [4]. Accordingly, primary epicardial access was selected.

**FIGURE 1:**
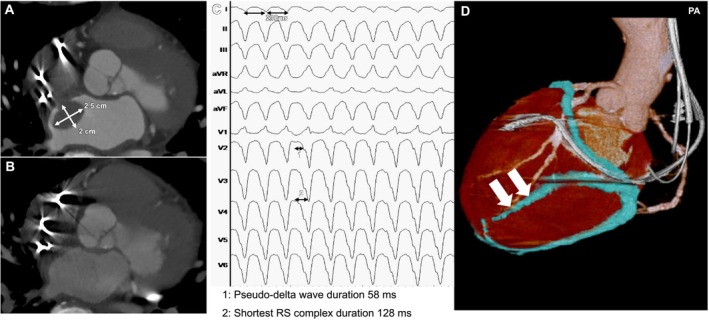
Clinical course and preprocedural evaluation before catheter ablation. (A) Preoperative CT showing a 2.5 × 2 cm left atrial mass consistent with a myxoma. (B) Postoperative CT showing complete resection of the left atrial myxoma following a robot‐assisted minimally invasive approach. (C) VT (cycle length, 290 ms) with a right bundle branch block pattern and a northwest axis showed a QRS morphology suggestive of an epicardial LV apical exit. The electrocardiogram fulfilled established epicardial criteria, including a pseudo‐delta wave of 58 ms (≥ 34 ms) and a shortest RS interval of 128 ms (≥ 121 ms). The intrinsicoid deflection time could not be assessed because no R wave was identifiable in lead V2. (D) Preoperative CT–based reconstruction of the CS was used for procedural planning, identifying the posterolateral branch (white arrow) as the target branch for intentional CS exit. CS, coronary sinus; CT, computed tomography; LV, left ventricular; VT, ventricular tachycardia.

A steerable sheath (Agilis; Abbott, St. Paul, MN, USA) introduced via the right femoral vein was advanced into the CS. Using preprocedural imaging as a reference (Figure 1D), a 0.014‐in. guidewire (Cruise; Asahi Intecc, Aichi, Japan) supported by a microcatheter (Prominent NEO; Tokai Medical Products Inc., Aichi, Japan) was advanced through a 5‐Fr Judkins Right catheter within the sheath and navigated into the posterolateral branch. After contrast injection through the microcatheter (Figure 2A), the guidewire was exchanged for a 0.014‐in. stiff guidewire (Astato 9–12; Asahi Intecc), and the distal segment of the branch was intentionally perforated to access the pericardial space (Figure 2B and Video S1). The microcatheter was then advanced into the pericardial space, and a total of 120 mL (approximately 2 mL/kg) of CO_2_ was manually injected at a slow rate (10–20 mL/min) under continuous invasive blood pressure monitoring. This volume was selected based on previous reports [2] and our institutional protocol to ensure adequate pericardial separation while monitoring for any signs of tamponade physiology.

**FIGURE 2:**
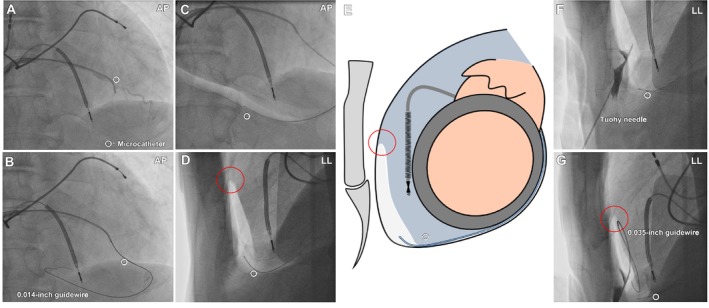
Fluoroscopic images of CO_2_ insufflation and percutaneous epicardial puncture. (A) Contrast injection through the microcatheter delineated the distal course of the posterolateral branch. (B) The distal segment of the branch was intentionally perforated using a 0.014‐in. stiff guidewire to access the pericardial space. (C, D) A total of 120 mL of CO_2_ was gradually injected through the microcatheter under continuous blood pressure monitoring before pericardial puncture. (C) AP view. (D) LL view showing partial restriction of CO_2_ distribution by adhesions over the anterior right ventricular surface related to prior cardiac surgery (red circle). (E) Schematic drawing before pericardial puncture showing partial restriction of CO_2_ distribution (red circle) by postoperative adhesions over the anterior right ventricular surface (see Figure 2D). (F) The suspected adhesion area was anatomically remote from the estimated VT exit site; therefore, percutaneous epicardial access was considered feasible. Under fluoroscopic guidance, subxiphoid pericardial access was obtained using an 18‐gauge Tuohy needle. (G) Despite the advancement of a 0.035‐in. guidewire to confirm secure pericardial access, the anterior right ventricular adhesions could not be separated with manipulation (red circle). AP, anteroposterior; CO_2_, carbon dioxide; LL, left lateral.

In the left lateral view, CO_2_ distribution was partially limited by adhesions over the anterior right ventricular surface related to prior cardiac surgery; nevertheless, a clearly delineated pericardial space was identified at the intended puncture site (Figure 2D,E and Video S2). The suspected adhesion area was anatomically distant from the estimated VT exit site. Therefore, percutaneous epicardial access was considered feasible.

Under fluoroscopic guidance, percutaneous subxiphoid pericardial access was obtained using an 18‐gauge Tuohy needle (B. Braun, Melsungen, Germany) (Figure 2F and Video S3).

A 0.035‐in. guidewire was advanced to confirm secure pericardial access. However, the anterior right ventricular adhesions could not be separated despite manipulation (Figure 2G and Video S4). An additional Agilis sheath was introduced into the pericardial space. Systemic anticoagulation was withheld until safe pericardial access was confirmed to minimize the risk of hemorrhagic complications during the puncture. CO_2_ was evacuated via the sheath without bleeding. Epicardial mapping was then performed during atrial lead pacing using a high‐density mapping catheter (OPTRELL, Biosense Webster, Irvine, CA, USA) with the CARTO 3 system (Figure 3A,B). Catheter manipulation induced clinical VT, which enabled the creation of a VT activation map (Figure 3C). The critical isthmus was identified on the epicardial surface and corresponded to an area with late potentials on substrate mapping. Epicardial radiofrequency ablation during VT terminated the tachycardia (Figure 4A), followed by additional ablation across the critical isthmus (Figure 4B). Complementary contralateral endocardial ablation achieved VT non‐inducibility with complete elimination of late potentials.

**FIGURE 3:**
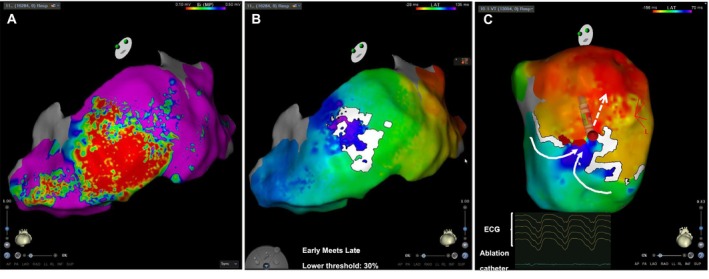
Epicardial electroanatomical mapping. (A, B) Epicardial mapping was performed during atrial lead pacing using a high‐density mapping catheter with the CARTO 3 system. (A) Voltage map (0.1–0.5 mV). (B) Local activation time map. A low‐voltage area was identified on the lateral aspect of the left ventricular apex, where late potentials were observed. (C) VT activation map showing the critical isthmus on the epicardial surface, which corresponds to the low‐voltage area showing late potentials on substrate mapping. Diastolic potentials were recorded with the ablation catheter (lower left portion), and radiofrequency application led to VT termination. VT, ventricular tachycardia.

**FIGURE 4:**
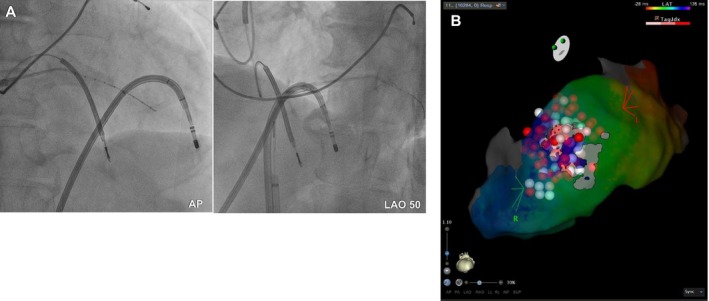
Ablation sites. (A) Fluoroscopic images showing the successful ablation sites (left: AP view; right: LAO view). (B) Final ablation sites displayed on a local activation time map. AP, anteroposterior; LAO, left anterior oblique.

No complications were found and the pericardial drain was removed after 2 days. No VT recurred during 6 months of follow‐up.

Epicardial ablation success depends not only on access but also on substrate reachability and mappability. This case shows that CO_2_ insufflation can inform pre‐procedural assessment of substrate accessibility beyond access facilitation alone. Restricted gas dispersion over the anterior right ventricular surface suggested localized post‐surgical adhesions. Indeed, pericardial mobilization in the corresponding area was unsuccessful despite catheter manipulation (Figure 2F). In contrast, adequate CO_2_ accumulation at the intended puncture site indicated preservation of the pericardial space.

Overall, we propose a simple decision framework based on CO_2_ distribution: (1) If CO_2_ distribution covers the target substrate estimated by the electrocardiographic data, as in the present case, which belongs to Brighton Adhesion Classification (BAC) 1a [5], percutaneous epicardial access is deemed safe; (2) if CO_2_ fails to disperse over the target substrate, suggesting epicardial adhesions shielding the critical isthmus, a surgical approach or an endocardial‐only strategy should be prioritized. The BAC framework assesses the severity of adhesions, while our case focused on the distance between the adhesion location and the targeted substrate. Although pre‐procedural computed tomography is useful for identifying calcifications or epicardial fat thickness, CO_2_ insufflation can demonstrate the absence of adhesions and provide functional information that static imaging modalities cannot offer. Note that CO_2_ insufflation may not detect subtle fibrosis that could still impact catheter maneuverability.

This case suggests that CO_2_ insufflation can serve as a useful adjunct for pre‐procedural assessment of pericardial adhesions in epicardial VT ablation. The technique may reduce procedural uncertainty and enhance safety in selected high‐risk patients, warranting further evaluation.

## Funding

The authors have nothing to report.

## Ethics Statement

This research was conducted according to the principles of the Declaration of Helsinki.

## Consent

The patient provided written informed consent for publication of the details of his case.

## Conflicts of Interest

K.Y. received speaker honoraria and lecture fees from Daiichi‐Sankyo, Johnson & Johnson/Biosense Webster, Medtronic Japan, Abbott Medical Japan, Japan Lifeline, and Kaneka Medix. The other authors have no conflicts of interest.

## Supporting information


**Video S1:** Fluoroscopic video showing intentional perforation of the distal posterolateral branch with a 0.014‐in. stiff guidewire via a microcatheter to access the pericardial space. Advancement of the guidewire outside the coronary venous silhouette indicates successful entry into the pericardial space.

**Video S2:** Cine fluoroscopic video obtained in the left lateral view shows restricted CO_2_ distribution over the anterior right ventricular surface (white arrow), suggesting pericardial adhesions related to prior cardiac surgery.

**Video S3:** Cine fluoroscopic video obtained in the left lateral view showing percutaneous subxiphoid pericardial access performed under fluoroscopic guidance with an 18‐gauge Tuohy needle, intermittently confirmed by small‐volume contrast injections.

**Video S4:** Cine fluoroscopic video obtained in the left lateral view showing failure to separate pericardial adhesions despite guidewire manipulation.

## Data Availability

The data that support the findings of this study are available from the corresponding author upon reasonable request.
